# Genetic sampling for estimating density of common species

**DOI:** 10.1002/ece3.3137

**Published:** 2017-07-03

**Authors:** Ellen Cheng, Karen E. Hodges, Rahel Sollmann, L. Scott Mills

**Affiliations:** ^1^ Daniel B. Warnell School of Forestry and Natural Resources University of Georgia Athens GA USA; ^2^ Wildlife Biology Program and Office of the Vice President for Research and Creative Scholarship University of Montana Missoula MT USA; ^3^ Department of Biology University of British Columbia Okanagan Kelowna BC Canada; ^4^ Department of Wildlife, Fish and Conservation Biology University of California Davis Davis CA USA

**Keywords:** density estimators, fecal pellets, noninvasive genetic sampling, snowshoe hare, spatial capture–recapture

## Abstract

Understanding population dynamics requires reliable estimates of population density, yet this basic information is often surprisingly difficult to obtain. With rare or difficult‐to‐capture species, genetic surveys from noninvasive collection of hair or scat has proved cost‐efficient for estimating densities. Here, we explored whether noninvasive genetic sampling (NGS) also offers promise for sampling a relatively common species, the snowshoe hare (*Lepus americanus* Erxleben, 1777), in comparison with traditional live trapping. We optimized a protocol for single‐session NGS sampling of hares. We compared spatial capture–recapture population estimates from live trapping to estimates derived from NGS, and assessed NGS costs. NGS provided population estimates similar to those derived from live trapping, but a higher density of sampling plots was required for NGS. The optimal NGS protocol for our study entailed deploying 160 sampling plots for 4 days and genotyping one pellet per plot. NGS laboratory costs ranged from approximately $670 to $3000 USD per field site. While live trapping does not incur laboratory costs, its field costs can be considerably higher than for NGS, especially when study sites are difficult to access. We conclude that NGS can work for common species, but that it will require field and laboratory pilot testing to develop cost‐effective sampling protocols.

## 1 INTRODUCTION

Estimating animal density is central to most wildlife management and conservation decisions, to assess trend, evaluate numeric responses to natural and anthropogenic disturbances, and measure population persistence. Capture–mark–recapture (CMR) models applied to live‐trap data have long been the standard for obtaining robust estimates of animal density (Pierce, Lopez, & Silvy, 2012; Pollock, Nichols, Brownie, & Hines, 1990). Unlike index‐based sampling (e.g., track or pellet transects), live‐trap data can be used to estimate detection probabilities from recapture histories of marked individuals, allowing statistically rigorous estimates of animal density. However, live trapping is invasive, is logistically daunting in remote areas, and can be difficult for some species.

Noninvasive genetic sampling (NGS) may be an effective alternative to live trapping, to obtain data amenable to CMR analysis (Lukacs & Burnham, 2005; Mills, Citta, Lair, Schwartz, & Tallmon, 2000; Schwartz, Luikart, & Waples, 2007). NGS can be used to construct CMR capture histories through individual genotypes “captured” from noninvasively collected samples of scat or hair. NGS coupled with CMR has been used to estimate densities for many uncommon or difficult‐to‐capture species, including jaguars (*Panthera onca* Linnaeus, 1758; Sollmann et al., 2013), grizzly bear (*Ursus arctos* Linnaeus, 1758; Ciucci et al., 2015), and wolverine (*Gulo gulo* Linnaeus, 1758; Mulders, Boulanger, & Paetkau, 2007). For species that are not easily trapped, NGS often yields larger sample sizes and is more cost‐effective than trapping efforts because scat and other sources of noninvasive DNA data are comparatively easy to collect (e.g., Hedges, Johnson, Ahlering, Tyson, & Eggert, 2013). The question we take up here is whether NGS can be as effective as live trapping for estimating density of relatively abundant and trappable species such as common raccoons *(Procyon lotor* Linnaeus, 1758*)*, Virginia opossums *(Didelphis virginiana* Kerr, 1792*)*, and snowshoe hares (*Lepus americanus* Erxleben, 1777); we use the latter as a tractable model for comparing live‐capture results and NGS data.

For many species, the fieldwork needed to collect noninvasive samples is cheaper and faster than live trapping, when both methods are used to obtain data over the same number of temporal sessions. But NGS gains an additional (and often large) field cost advantage, especially with difficult‐to‐access sites, when the capture history for CMR analyses is built from DNA data collected during a single site visit. With live trapping, animals must be released from traps between each sampling session. In contrast, during a single NGS sampling session lasting days to weeks, animals can leave their genetic signatures at multiple “traps,” generating spatial capture histories analogous to the temporal capture histories from live trapping. Spatial capture–recapture (SCR; Efford, 2004; Borchers & Efford, 2008) models are extensions of CMR that can be used for analyzing spatial capture histories from single‐session surveys.

Obtaining reliable population estimates with NGS depends not only on the number of genetic samples collected from the field, but also on the proportion of those samples that yield correct genotypes. To minimize genotyping errors and laboratory costs, field sampling must be designed to ensure genetic samples are fresh. Even then, two types of genotyping errors—false alleles and allelic dropout—can occur with the low quality and quantity of DNA available from NGS (Taberlet, Waits, & Luikart, 1999). Both error types can falsely inflate the number of unique individuals identified, leading to density estimates that are biased high (Waits & Leberg, 2000). These genotyping errors can be reduced through repeat amplification of genetic samples and reanalysis of samples with highly similar genotypes (Taberlet et al., 1996). Alternatively, density may be underestimated if the number and variability of molecular markers used in genotyping is inadequate to distinguish individuals, which may require sampling more loci to differentiate between even closely related individuals (Mills et al., 2000), again raising laboratory costs.

Laboratory costs for NGS studies are greater for common than rare species, because costs accrue based on the number of samples genotyped (Lukacs & Burnham, 2005; Marucco, Boitani, Pletscher, & Schwartz, 2011). Thus, NGS surveys for density estimation are primarily used for rare or difficult‐to‐capture species, where the method provides obvious advantages over live trapping. Few studies have assessed the reliability and cost of NGS surveys for common, readily catchable animals. To address this gap, we evaluated the cost‐effectiveness of a single‐session NGS approach for estimating density of snowshoe hares, a common species in boreal forests of North America. We asked three questions to determine whether and when this NGS approach is a viable alternative to live trapping: (1) Can we obtain sufficient genetic samples from the field while meeting single‐session CMR assumptions of population closure and no un‐modeled capture heterogeneity? (2) Can we achieve high genotyping success? (3) Are density estimates from live trapping and NGS comparable? We also consider the cost‐effectiveness of the two methods for obtaining analyzable capture histories.

## 2 MATERIALS AND METHODS

### 2.1 Study species

**Figure 1:**
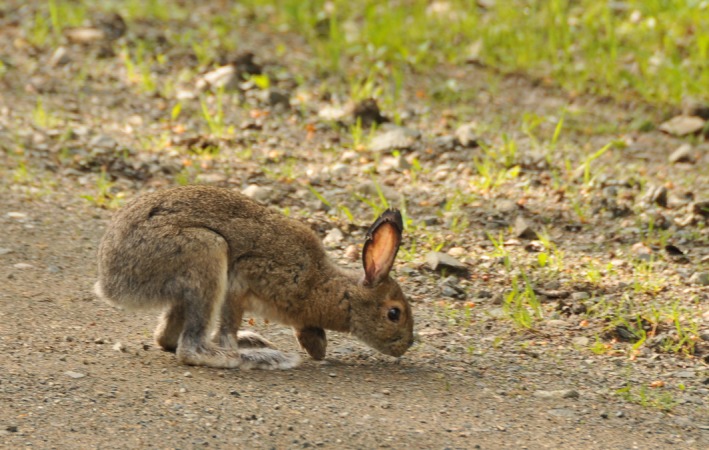
Snowshoe hare (*Lepus americanus* Erxleben, 1777). Photograph credit: Karen E. Hodges

Snowshoe hares are medium‐sized herbivores widespread in montane and boreal forests of North America (Figure 1). In Canada and Alaska, the species' northern range, population densities may reach six hares per ha during the high phase of 10‐year population cycles (Hodges, 2000a). In these northern boreal forests, population sizes of Canada lynx (*Lynx canadensis* Kerr, 1792) and other major predators closely track the population cycles of snowshoe hares. In the contiguous United States, snowshoe hare populations are less cyclic and densities rarely exceed 2.7 hares per ha (Hodges, 2000b). Snowshoe hares occupy overlapping home ranges that can cover 1.6–10.2 ha during the year. They are an important game species in many regions where they occur.

### 2.2 Study area

This work was implemented in summers 2006 and 2009 at nine 20‐ha sites in two study areas in Montana. Three sites were in Glacier National Park (Cheng, Hodges, & Mills, 2015) and six in Flathead National Forest west of Glacier NP (Hodges & Mills, 2008), both areas of more extensive sampling for other research questions. Five sites were used in a pilot study, and five sites (including one from the pilot study) were used in a field survey. We selected the sites to reflect a range of hare densities and forest types. Average monthly high temperatures for the study season ranged from 24*°* C to 27*°* C. Average monthly total precipitation ranged from 4.3 to 8.9 cm.

### 2.3 Optimizing an NGS field protocol (pilot study)

First, we determined whether our NGS approach could yield sufficient fresh samples for reliable CMR‐based population estimates. Because snowshoe hares deposit >500 pellets per day (Hodges, 1999), collection from the forest floor would be difficult in terms of determining which pellets and how many to collect; aging pellets is subjective, pellets might be missed, and defining independent samples would be tricky. Instead, we established specific sampling stations where we deployed baited 0.5‐m^2^ ground cloths that were left in the field for several days. Ground cloths ensured we obtained fresh hare pellets, which helped both with DNA amplification and with meeting the CMR assumption of “closure” of the population during sampling. Each 20‐ha (400 × 500 m) study site was divided into an 8 × 10 grid with 50‐m spacing between plots, for a total of 80 NGS plots per site. This sampling design echoed our survey method for live trapping snowshoe hares.

A hare can deposit multiple pellets during a single visit to an NGS plot. Therefore, on each NGS plot, only pellets from different hares (determined by pellet genotypes) can be considered independent captures for CMR analyses. In optimizing our NGS field protocol, we therefore sought to increase the number of NGS plots with pellets, rather than to increase the number of pellets collected per plot. Independent captures from different plots could be increased by extending the number of sampling days, using attractive baits, or increasing the number of ground cloths per site. The first two options were evaluated during a pilot study, described below. The latter was assessed during our field survey, by doubling the number of sample plots at a subset of sites.

#### 2.3.1 Pellet accumulation pilot study

In summer 2006, we pretested survey methods at five pilot study sites. We examined impacts of sampling duration by counting how many NGS plots at each site had pellets after 1–5 days of sampling. To compare bait efficacy, at each site we randomly assigned one of three bait types (apples, oats, alfalfa) to each plot. We used a Kruskal–Wallis rank sum analysis to test for differences among bait types in the percentage of plots that accumulated pellets.

#### 2.3.2 Pellet decomposition pilot study

Genotyping success declines with the time samples are left in the field, because DNA degradation increases with temperature, moisture, and exposure to ultraviolet radiation (Murphy, Kendall, Robinson, & Waits, 2007). An optimal NGS sampling duration would be long enough to collect many pellets, but short enough to ensure high genotyping success. Therefore, simultaneous with determining pellet accumulation rates in the field, we conducted a study of how quickly hare pellet DNA degrades with field exposure.

Degraded DNA can manifest as low PCR amplification success or high genotyping error rates. Estimating genotyping error rates often relies on comparison with reference genotypes from high‐quality DNA sources (Broquet & Petit, 2004). To quantify genotyping error rates for different‐aged pellets, we collected an ear tissue sample (reference genotype) and pellets from each of 18 snowshoe hares captured at two pilot study sites. The pellets were obtained from the floors of live‐traps that contained the captured hares. At the time of collection, these pellets could have been up to 12 hrs old (traps were open overnight). We transferred the pellets to a forest near our base camp. At 0, 2, 4, and 6 days postcapture, we selected up to three pellets from each hare's pellet pile for genetic analysis. Once selected, pellets were stored in 95% alcohol in a −20*°*C freezer until extraction, which occurred within 6 months of collection.

We used Qiagen DNeasy Blood & Tissue Kits to extract DNA from the 18 hare tissue samples. The pellet samples were extracted with QIAamp DNA Stool Mini Kits, in a separate laboratory at University of Montana designated exclusively for low quality DNA samples collected noninvasively. All samples were genotyped at eight highly variable microsatellite loci originally developed in the European rabbit, *Oryctolagus cuniculus* Linnaeus, 1758, and successfully used with snowshoe hares (Burton, Krebs, & Taylor, 2002; Cheng, Hodges, Melo‐Ferreira, Alves, & Mills, 2014; Schwartz, Luikart *et al*, 2007; Schwartz, Pilgrim, McKelvey, Rivera, & Ruggiero, 2007). PCR amplifications were run on an ABI 3130xl Genetic Analyzer (Murdoch DNA Sequencing Facility; Missoula, MT) and scored with GENEMAPPER v. 3.7 (Applied Biosystems Inc., Foster City, CA, USA). We manually checked all microsatellite genotypes to confirm allele calls. Sample extraction and PCR conditions are described in Cheng et al. (2014). PCR amplifications of the eight loci were combined into three multiplex reactions, and each tissue and decomposition pellet sample was amplified four times across all loci.

For each pellet age class, we calculated nonamplification as the proportion of PCR attempts in which a sample locus did not yield any genotype. Nonamplification rates were averaged across all loci, samples and PCRs. Using the corresponding tissue samples as reference genotypes, we calculated allelic dropout rates, false allele rates, and base shift error rates averaged across all loci, samples and PCRs for each pellet age class (Program GIMLET v1.3.3; Valiere, 2002). Allelic dropout was observed when a heterozygote was typed as a homozygote. A false allele occurred when slippage during PCR generated an additional erroneous allele. Base shift errors were small shifts in allele size—typically a one‐base pair increase or decrease. Because calculations used different denominators, error rates could not be summed to yield total error rate for each pellet age class (Broquet & Petit, 2004).

### 2.4 Collecting live‐trap and NGS data (field survey)

After optimizing an NGS protocol based on the pilot study, we conducted live trapping and NGS at two sites in Glacier NP in 2006 and at three sites in Flathead National Forest in 2009. At each site, live trapping and genetic sampling occurred sequentially within 2 weeks and in random order (i.e., live trapping then genetic sampling or the reverse). Significant differences in population estimates are attributable to differences in the survey methods rather than to changes in hare density within this short time.

#### 2.4.1 Live trapping

At each site for the field survey, we placed 80 Tomahawk live‐traps in the same 8 × 10 grid configuration described for the pilot study. Each site was trapped for three to five nights. Traps were opened every evening and baited with apple and alfalfa and then checked the following morning. Captured hares were weighed, sexed, and ear tagged. We used sterile 3‐mm biopsy punches to collect a small piece of ear tissue from each hare for genetic analyses. All hare handling was approved by the University of Montana's IACUC. Ear tissue samples were stored in silica gel until return from the field, at which point they were frozen to −20°C.

#### 2.4.2 Noninvasive genetic sampling

Noninvasive genetic sampling followed a refined protocol developed from our pilot work. At each of the 80 grid points, we baited a 0.5‐m^2^ ground cloth with two to three commercially produced alfalfa cubes that were 2.5 to 5 cm per side. We returned 4 days later to collect all pellets that had accumulated on the ground cloths. At the three Flathead sites, we also tested the efficacy of using 160 cloths, placing the additional cloths halfway between the main sampling plots.

### 2.5 Genotyping and error‐checking genetic samples

We genotyped all tissue genetic samples collected from live‐trapped hares. For each tissue sample, a “consensus genotype” was confirmed when two independent PCR amplifications yielded the same genotype. From confirmed tissue genotypes, we estimated allele frequencies, heterozygosities, and probability of identity (PID and PID_sib_) by locus, using Program GIMLET. PID is the probability that two individuals (or siblings, for PID_sib_) drawn from the population have identical confirmed genotypes. The PID and PID_sib_ were determined by multiplying the eight locus‐specific estimates.

Genotyping all pellets collected by NGS would have been expensive and likely redundant. Multiple pellets from one hare's visit would be quite possible (Hodges & Sinclair, 2005). But if the multiple pellets on a single plot were instead from different hares, we would lose important capture information if we genotyped only one pellet per plot. As a compromise to minimize laboratory costs while maximizing potential capture rates, we genotyped up to four randomly selected pellets from each NGS plot. When plots had four or fewer pellets, all were genotyped.

For each NGS pellet selected, we determined a “consensus genotype” based on a stringent error‐checking protocol. Pellet samples with <40% amplification success, across eight microsatellite loci, in the first two PCR runs were omitted from further analysis. We then used a three‐stage approach and mismatch comparisons, modified from Waits and Paetkau (2005), to confirm a consensus genotype for each sample. A sample was designated a confirmed homozygote at a locus if it amplified as a clear homozygote in at least four PCRs with no discrepancies, and as a confirmed heterozygote if each allele amplified clearly in at least two PCRs with no discrepancies. If a genotype was confirmed at all loci, or if the eight‐locus genotype matched that of another genetic sample collected from the same site, the eight‐locus genotype was the sample's consensus genotype. Each sample was amplified up to six times per locus.

### 2.6 Comparing live‐trap and NGS density estimates from field surveys

We applied maximum‐likelihood spatial capture–recapture models, implemented in the R package “secr” (Efford, 2016), to estimate densities separately from live‐trap and NGS data for each study site. For SCR analysis, we assumed animal activity centers were distributed according to a homogeneous point process and detection probability followed a half‐normal function. NGS plots were modeled as single occasion proximity detectors, which allowed individuals to be captured at multiple detectors during a survey occasion, but at each detector an individual was counted only once. We modeled live‐traps as multicatch traps, a substitution that is minimally biased (Efford, 2016), because a single‐catch likelihood is not available with maximum‐likelihood estimation in “secr.” For live‐trap data, we used Akaike's Information Criterion for small sample sizes (AICc) to rank support for three detection models: The null model, a model that included capture‐related behavioral effects (e.g., trap shyness), and a two‐class mixture model allowing for two groups with different detection probabilities (e.g., males vs. females or adults vs. juveniles). For the NGS data, we used AICc to rank support for the null and two‐class mixture models. The behavioral model was not applicable because there was only one survey occasion. We set an integration buffer width of 600 m, which exceeds the minimum recommended width of three times the σ parameter (a spatial scale parameter that describes how quickly detection probability declines as the distance between a trap and an animal's activity center increases) estimated from the data (Royle, Chandler, Sollman, & Gardner, 2013).

To examine how density estimates are influenced by the number of pellets genotyped per plot, we analyzed data from 500 iterations each of computer‐generated subsampling of one or two pellets per plot, randomly selected from the four‐pellet NGS dataset. We also tested three and four pellets per plot, but results were very similar to two pellets per plot, so are not reported. For each level of pellet sampling, the median of the density estimates and the median of the 95% confidence intervals from estimable iterations (capture histories with at least one recapture) were compared to estimates from live‐trap data. When there are no recaptures, SCR model parameters are not identifiable.

### 2.7 Comparing live trapping and NGS through simulation

To compare the accuracy and precision of estimates from live‐trap versus NGS methods, for different hare densities and with different assumptions about the movement distances of hares, we simulated 500 iterations of 135 scenarios modeled after our study system. Using SCR model formulation, we simulated five sampling approaches: for live trapping, a single‐trap detection model with four survey occasions, using the 80‐plot trap grid of our empirical study (8 × 10 grid); for NGS, a proximity detection model with one survey occasion, using either an 80‐ or 160‐plot grid and either one or two pellets sampled per plot. For each sampling approach, we simulated three hare densities (0.2, 1.0, and 1.8 hares per hectare) × three detection probabilities at activity center (g0 = 0.05, 0.10, and 0.15) × three levels of σ (20, 50, and 80 m) with a half‐normal detection function. The SCR parameters used in this simulation spanned the range of values estimated from 25 site‐years of hare data collected from our long‐term research in the Flathead NF (LS Mills & KE Hodges, unpublished data).

The 500 simulated capture histories for each scenario were analyzed as described above for the empirical data. We calculated the following summary metrics to compare density estimates from the simulated live‐trap versus NGS approach:

1. Proportion of estimable iterations, measured as the proportion of 500 iterations with at least one recapture. Only estimable iterations were included in other summary metrics. If fewer than 10% of iterations were estimable for any simulated scenario, summary metrics were not calculated.
2. Root mean square error, a combined measure of bias and variance, calculated as $\sqrt{\frac{\sum {\left(\hat{D}-D\right)}^{2}}{n}}$, where $\hat{D}$ is estimated density, *D* is true density, and *n* is the number of iterations.
3. Median coefficient of variation across iterations, with the coefficient of variation for each iteration calculated as the estimated standard error divided by estimated density.
4. Confidence interval coverage, which was the proportion of iterations in which the 95% confidence interval included the simulated (true) parameter value.

## 3 RESULTS

### 3.1 Optimizing an NGS field protocol (pilot study)

**Figure 2:**
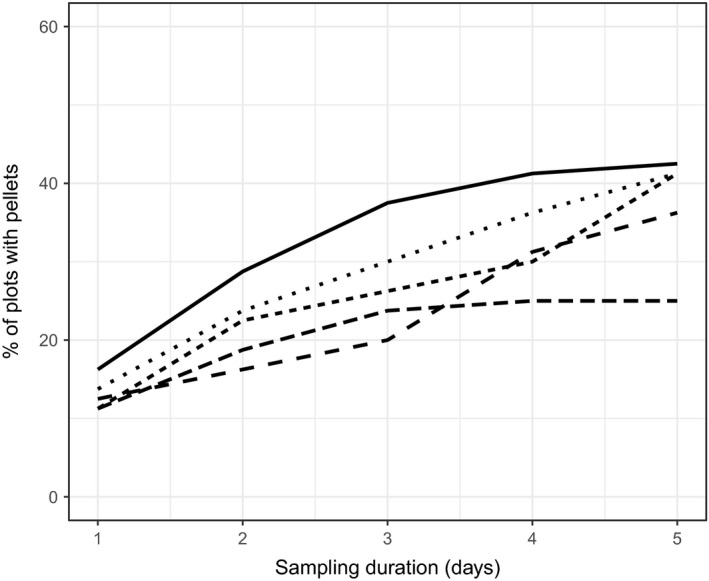
Accumulation of snowshoe hare pellets on NGS sampling plots as a function of sampling duration. Each line represents a different 20‐ha pilot site

**Figure 3:**
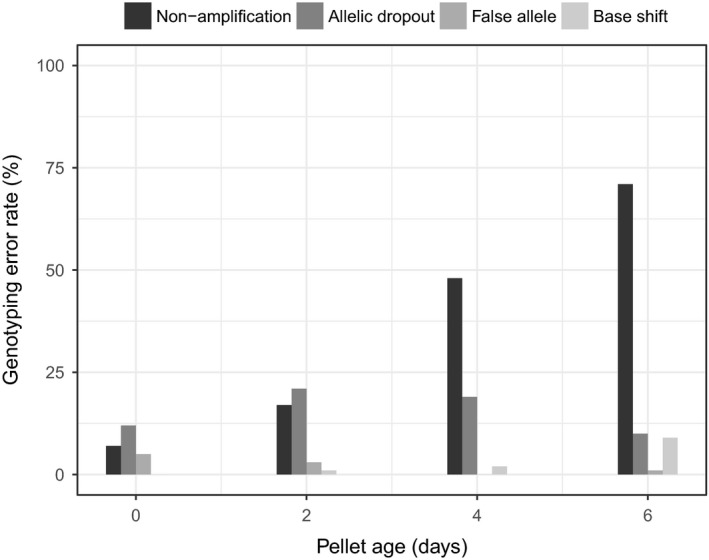
Per‐locus genotyping error rate as a function of pellet age. Error rates are averaged across all loci, samples and PCRs. Nonamplification is when a sample‐locus does not yield any genotype. Allelic dropout occurs when a heterozygote is typed as a homozygote. A false allele is an additional erroneous allele. Base shift errors are one‐ or two‐base pair shifts in allele size

After 1 day of sampling, 13 ± 2.1% (*SD*) of plots at pilot study sites had hare pellets (Figure 2). The number of plots with pellets increased most rapidly during the first 2 days of sampling. By the fifth day of sampling, 37.2 ± 7.2% of plots had at least one pellet. Per‐locus genotyping error rates increased with the number of days pellets were left in the field (Figure 3). Averaged across the eight loci, only a small percentage of 0‐day‐old pellets did not amplify at a locus (7%), but 71% of 6‐day‐old pellets did not amplify at a locus. When genotypes did amplify at a locus, allelic dropout was the most common type of genotyping error (Figure 3).

The three baits (apples, oats, and alfalfa) were similar at attracting hares to sampling plots (χ^2^ = 1.31, *df *= 2, *p* = .52). Alfalfa was easiest to handle and minimized disturbance to plots (most commonly, deer attracted to apples). Our final NGS protocol used 4 days of sampling with alfalfa as bait. The nonamplification rate for 4‐day‐old pellets was almost 50%, but a majority of the pellets collected after 4 sampling days would be <4 days old.

### 3.2 Collecting live‐trap and NGS data (field survey)

From 119 live‐trap captures of snowshoe hares across the five study sites, we identified 72 unique individuals (Table 1). The average number of live‐trap captures per site was 23.8 (*SD* = 14.5), and the average number of unique individuals per site was 14.4 (*SD *= 10.0). We collected 488 snowshoe hare pellets from ground cloths (Table 2). Eighty percent of sampling plots had no hare pellets, but variation among sites was high. On average, 7% of sampling plots at each site had one pellet; 4%, two pellets; 2%, three pellets; and 7% had four or more pellets. At the three Flathead sites, the proportion of plots with pellets was similar when considering the 80 main plots versus all 160 plots. One site (Flathead 2) had 2 days of rain during the 4‐day pellet accumulation period, but we did not observe a clear negative impact of rain on pellet numbers or PCR success (i.e., some other sites without rain had fewer pellets or lower PCR success than Flathead 2).

**Table 1:** Live‐trap hare captures

Site	Number of trap nights	Total number of captures	Number of unique hares captured
Glacier1	5	40	30
Glacier2	3	12	7
Flathead1	4	9	8
Flathead2	4	38	19
Flathead3	4	20	8

**Table 2:** NGS pellets collected and genotyped. For Flathead sites, the first value is based on 80 NGS plots; second, 160 NGS plots. We genotyped up to four randomly sampled pellets per plot. From a subset of genotyped pellets, we were able to obtain reliable consensus genotypes for individual identification (“% estimable genotypes”)

Site	% of plots with pellets	Number of pellets collected	Number of pellets genotyped	% estimable genotypes	Number of unique hares
Glacier1	45	138	87	91	32
Glacier2	6	26	17	70	5
Flathead1	13/10	19/37	19/33	79/88	8/10
Flathead2	18/18	70/138	29/60	66/68	11/17
Flathead3	19/19	102/149	34/72	62/68	13/21

### 3.3 Genotyping and error‐checking genetic samples from field surveys

We obtained and successfully genotyped genetic samples from 85% (*N* = 61) of the 72 live‐trapped hares. The 11 missing genetic samples were primarily due to hares escaping before samples could be obtained. Based on tissue samples, average observed heterozygosity for the eight loci was 0.71 (*SD* = 0.19; Table 3). The number of alleles per locus ranged from four (SOL33) to 29 (SOL30). Overall PID, calculated as the product of the locus‐specific values, was 1.8 × 10^−10^ and PID_sib_ was 8.9 × 10^−4^.

**Table 3:** Microsatellite diversities and probability of identity, by locus

Locus	A	Ho	PID	PIDsib
7L1D3	7	0.63	0.18	0.48
SAT02	26	0.86	0.01	0.29
SAT12	6	0.81	0.11	0.42
SAT13	6	0.67	0.20	0.49
SOL33	4	0.33	0.46	0.70
SAT16	8	0.62	0.11	0.43
SOL08	9	0.87	0.06	0.36
SOL30	29	0.87	0.00	0.28

A, number of different alleles; Ho, observed heterozygosity; PID, probability of identity; PIDsib, probability of identity for siblings.

Across the five study sites, 55% (*N* = 269) of the 488 pellets collected by NGS were genotyped, of which 210 (78%) amplified and yielded consensus genotypes. We conducted an average of 4.6 PCR runs per pellet. After error checking, 87% of pellet samples had genotypes that could be matched to another pellet or to a tissue genotype from a hare live‐trapped at the same site. The consensus genotypes for three pellets (of 210) differed from another sample at only one locus. After additional independent PCRs, we concluded the consensus genotypes for these pellets represented unique individuals. For 3% of sampling plots, we confirmed pellet genotypes for two unique hares; for 1.1% of plots, three unique hares.

### 3.4 Comparing live‐trap and NGS density estimates

**Figure 4:**
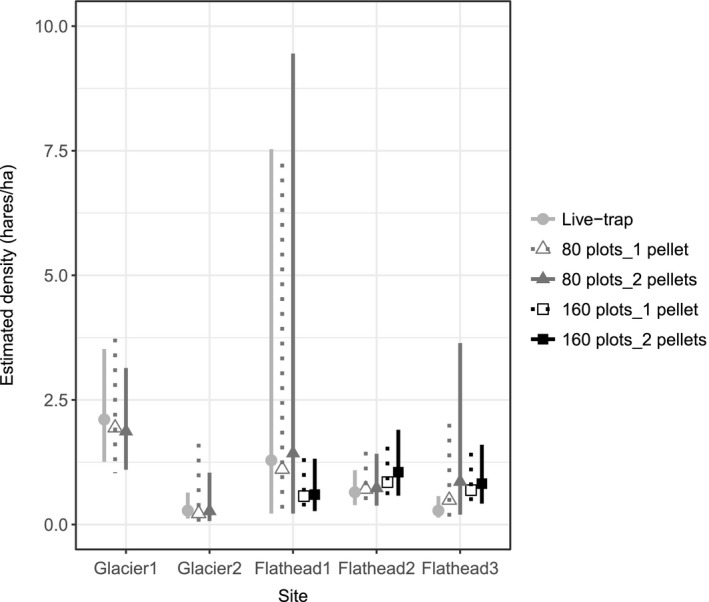
Snowshoe hare density estimates and 95% confidence intervals, based on spatial capture–recapture analysis of live trapping and noninvasive genetic sampling (NGS) at five sites. NGS results are shown for two sampling densities (80 and 160 plots per site) and computer‐generated subsampling of one or two pellets per NGS plot, each for 500 iterations. We present the median density estimates and median confidence intervals of estimable iterations

At all sites, for both live‐trap and NGS data, the null model was the highest ranked SCR model, with at least 75% AICc weight. The 95% CI for hare density estimates overlapped substantially among methods (Figure 4). Confidence intervals were usually largest for 80‐plot NGS sampling. Unusually large 95% CI's corresponded with recapture rates <20% (Table 4). Sampling one versus two pellets per plot for genotyping had little influence on density estimates (Figure 4).

**Table 4:** At each site, the % of unique hares recaptured. With live trapping, a hare is recaptured if it is caught on more than one trap night. With single‐survey NGS, a hare is recaptured if its genotype is confirmed from pellets collected from more than one NGS plot. For NGS, we present the median value of estimable iterations from subsampling one or two pellets per plot

Site	Live‐trap	80 plots, 1 pellet	80 plots, 2 pellets	160 plots, 1 pellet	160 plots, 2 pellets
Glacier1	30	31	40	NA	NA
Glacier2	57	33	40	NA	NA
Flathead1	12	14	12	30	50
Flathead2	63	20	27	47	41
Flathead3	75	12	9	40	42

No SCR detection parameters were consistently higher or lower across sites for a particular sampling method (live‐trap vs. NGS). Excluding Flathead2 80‐plot NGS, the average g0 (detection probability at activity center) was 0.09 and ranged from 0.03 to 0.24 for all sites and methods. The parameter σ averaged 57.6 m and ranged from 22.1 to 98.4 m. SCR detection parameter estimates for Flathead2 80‐plot NGS were unusual, with a very high g0 of 1.0 and small σ estimates (18.1–19.9), yet still yielding density estimates similar to live trapping.

### 3.5 Comparing live trapping and NGS through simulation

**Figure 5:**
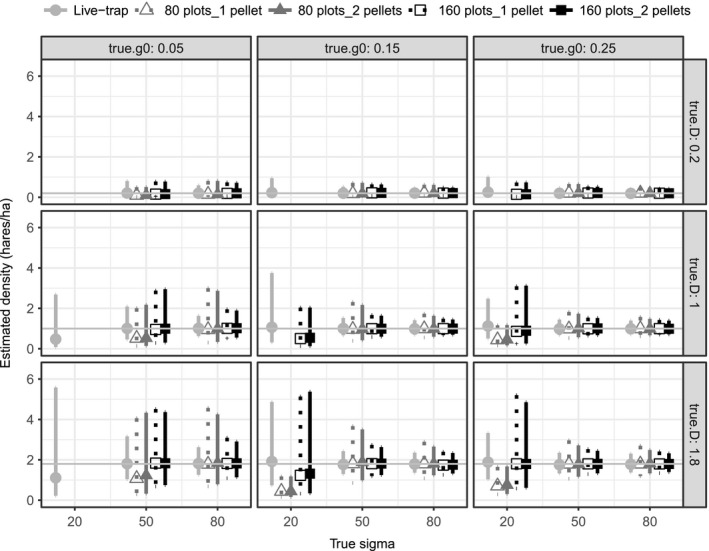
Snowshoe hare density estimates and 95% confidence intervals, from simulations based on our snowshoe hare study system. For each of five sampling approaches (figure legend), we simulated three hare densities (0.2, 1.0, and 1.8 hares per hectare, in figure rows), three detection probabilities (g0 = 0.05, 0.10, and 0.15, in figure columns), and three levels of sigma (20, 50, and 80 m, on *x*‐axis in figure cells). For each simulated scenario, we present the median density estimates and median confidence intervals of estimable iterations. If <10% of the 500 iterations were estimable, results are blank

In simulations, live trapping and 160‐plot NGS produced more accurate and precise density estimates than did 80‐plot NGS. As with the field data, the number of pellets genotyped had little influence on estimates. At the smallest σ (20 m), hare density often could not be estimated, was biased low, or had a large 95% CI (Figure 5). Density estimates were also poor when low detection (g0 = 0.05) resulted in few individuals captured or a low proportion of recaptures, even if simulated hare densities were moderate to high (Appendix S1). Regardless of survey method, when unique captures exceeded 20 individuals and at least 20% of captured animals were recaptured, density estimates were generally unbiased and close to true values (Appendix S1).

Root‐mean‐square error and coefficient of variation were often smaller with live trapping and 160‐plot NGS compared to 80‐plot NGS, except at the smallest σ simulated (Appendix S2). Root‐mean‐square error declined as density declined, and as g0 or σ increased. Coefficient of variation declined as density, g0, or σ increased. Confidence interval coverage was close to 95% except for 80‐plot NGS at the lowest g0 or smallest σ.

## 4 DISCUSSION

Single‐session NGS was a viable alternative to multiple‐session live trapping for estimating densities of snowshoe hares under a range of field and simulation conditions. When detection probability was very low or hare movements limited, additional sampling plots were required for NGS to yield density estimates comparable to live trapping. Increasing the density of NGS plots at a site is relatively easy and cheap, and an important benefit of NGS is that all genetic samples can be collected in a single site revisit, compared to the multiple survey nights required with live trapping. NGS density estimates should also be improved by increasing the number of survey sessions, but this option may be more expensive than increasing sampling plots and was not evaluated in this study.

### 4.1 Optimizing an NGS protocol

Studies addressing effects of environmental exposure on DNA degradation have suggested that NGS samples should be collected within a few days to a week of deposition, but in some cold and dry environments, samples up 1 month old still had reasonable (>60%) genotyping success (Murphy et al., 2007; Piggott, 2004; Stetz, Seitz, & Sawaya, 2015). We identified an optimal NGS sampling duration of 4 days for snowshoe hare pellets. Our 80% genotyping success was relatively high for NGS (Marucco et al., 2011). The proportion of sampling plots with pellets increased steadily over the 4 days. With a sampling density of 160 plots per site, this duration usually yielded sufficient samples for reliable population estimates. These results are specific to our study species, sampling design, and survey conditions (e.g., timing of sampling and weather), so we recommend that other researchers conduct similar presurvey testing prior to conducting NGS population estimates. For example, sampling duration may need to be reduced for surveys conducted in warmer and wetter months or sites, due to lower genotyping success.

Nonamplification rates increased rapidly with pellet age over 4 days of sampling. Genotyping success could be further improved by limiting sampling duration to 2 or 3 days, while increasing the density of sampling plots to maintain similar capture and recapture numbers. A shorter sampling duration could reduce per‐sample laboratory costs, as fewer PCR runs could be required to obtain reliable consensus genotypes. Eliminating the most problematic loci (those with the lowest amplification success and highest error rates) could also reduce costs while potentially improving density estimates, provided the remaining loci are sufficiently variable to minimize error due to the “shadow effect” (Mills et al., 2000).

### 4.2 Comparing density estimates among survey methods

In field and simulation studies, we compared the accuracy and precision of density estimates from live trapping, 80‐plot NGS, and 160‐plot NGS. The number of pellets genotyped per NGS plot had little influence on density estimates. This finding is not surprising, as multiple pellets on a single plot often arose from just one individual; we focus on one‐pellet results hereafter.

Previous studies on multiple‐session CMR surveys demonstrated that when trap spacing is more than twice the value of σ, SCR estimates may be poor (Sollmann, Gardner, & Belant, 2012; Sun, Fuller, & Royle, 2014). Our simulations corroborated these findings. At the lowest σ simulated (20 m), trap spacing exceeded individual hare movement distances, recaptures were rare, and all methods performed poorly. For higher σ, live trapping and 160‐plot NGS density estimates were unbiased and precision was comparable. However, density estimates from 80‐plot NGS were sometimes still biased and almost always had larger 95% CI's than the other methods did.

In multiple‐session live trapping, a recapture can occur at the same trap on different trap nights, although that kind of recapture alone does not provide the spatial information necessary to estimate the detection parameters in a SCR model. With single‐session NGS, a recapture occurs only when an animal is detected at different plots. This distinction may explain why a higher density of NGS plots (160 instead of 80) was required to achieve density estimates comparable with live trapping at low σ.

Differences in recapture and initial capture rates underpinned differences in density estimates among survey methods, most evident at low g0 or σ. Density estimates were highly variable, sometimes greatly exceeded true simulated densities, and had large 95% CI's when there were few recaptures (<~20%). In simulations, recapture rates were almost always lower for 80‐plot NGS than for live trapping or 160‐plot NGS. This result is intuitive, as live trapping occurred over multiple occasions, and sampling density in the 160‐plot design was twice as high as in the 80‐plot scenario. Field results were consistent with these findings—the three cases with large 95% CI's (one live‐trap and two 80‐plot NGS) had <20% recaptures.

Simulations also identified a target threshold for initial captures. When fewer than ~20 unique individuals were captured (unless low captures were due to low densities), estimated density was frequently biased low. Similarly, White, Anderson, Burnham, and Otis (1982) recommended at least 20 unique individuals and 30% capture probability for reliable CMR estimates.

### 4.3 Cost‐effectiveness of live trapping versus NGS

When the primary variable of interest is a population estimate, single‐session NGS may be a cost‐effective alternative to live trapping, even for common species like snowshoe hares. NGS is especially advantageous when study sites are difficult to access, because noninvasive genetic samples are often much easier and cheaper to collect in the field compared to live‐trap data.

Single‐session NGS survey costs entail two site visits (deployment and collection), field supplies, and laboratory costs. It is difficult to reduce the field costs any further; the primary variables that can be adjusted are the number of cloths deployed (in USD, ~$15 for 80 cloths; $30 for 160 cloths) and the duration in the field. In the laboratory, there are significant cost and data‐quality trade‐offs concerning pellet freshness, numbers of attempted amplifications, and re‐runs to establish consensus genotypes. All of these costs increase as the number of pellets to analyze increases, and that number is a function of actual hare density and the field sampling design.

With our optimized 160‐plot NGS protocol, the per‐sample cost covering laboratory labor and genetic supplies in this study was $42. Using this protocol, estimated laboratory costs for our Flathead sites ranged from $672 (Flathead1: 16 plots with pellets X $42 per pellet) to $1260 (Flathead3: 30 plots with pellets × $42 per pellet). At our highest density site (Glacier1), we only deployed 80 NGS plots, but assuming the proportion of plots with pellets would be similar for 160 plots, the estimated laboratory cost would have been $2940 (70 plots with pellets × $42 per pellet).

Live trapping surveys have no laboratory costs but entail higher field costs, because of the high number of site visits (our protocol involved opening traps at night and checking traps the following morning, for three to five nights of trapping) and more time and labor required to deploy live‐traps than NGS cloths. In our case, the genetic surveys would take about 8–12 person hours in the field (two people× two visits × two to three hours per visit), whereas trapping would take anywhere from 30 person hours on our easiest sites (nine person hours to set out traps, nine person hours to collect them, three evenings × two person hours to set traps, and three mornings × two person hours to check traps) to 80–1120 hrs for harder sites (more brush, deadfall, hill, or further from roads), sites with more hares to handle, or for more nights of trapping. If surveying backcountry study sites, the additional time and expense of overnight stays for live trapping could be even more considerable. At an hourly wage of $10, the costs would become $80 to $120 for the field time for the genetic survey, and anywhere from $300 to $1200 for live trapping each site, plus all the additional gasoline for the repeat site visits. These simple cost estimates suggest that when hares are very abundant or laboratory costs expensive, live trapping may be more cost‐effective, but that harder sites to navigate or sites with few hares may be more efficiently sampled via the NGS pellet protocol.

In wildlife studies, issues other than cost and the reliability of population estimates are often important for evaluating the cost‐effectiveness of survey methods. If studies require the additional data that live‐captures afford (e.g., age or body mass information, opportunities for radio‐collaring, or blood or tissue biopsies for disease work), then this approach to population estimation makes more sense than employing an NGS approach. On the other hand, NGS has the huge advantage of being noninvasive and less disruptive to wildlife populations, and often less visible in the field, which is advantageous in areas with many tourists, such as national parks.

## 5 CONCLUSIONS

Surprisingly few comparisons have been made between traditional trap‐based and noninvasive estimates of density, and to our knowledge, none have asked whether noninvasive genetic methods can be cost‐efficient for surveying relatively common and easily trappable species such as snowshoe hares. These questions are increasingly relevant because the downsides of noninvasive genetic sampling have been rapidly decreasing with improved laboratory and analytical techniques.

Our comparison of NGS and live trapping for snowshoe hares shows that NGS could indeed be viable. Our pilot work was essential for determining an appropriate trade‐off in the collecting period between acquiring more pellets and avoiding excessive pellet degradation. We also found that increasing the sampling density (from 80 to 160 cloths per site) greatly improved NGS results. Both live trapping and NGS methods suffer when recapture rates are low. We encourage researchers contemplating an NGS approach to calculate a cost comparison between methods for their study system; NGS does have much lower field costs, but those need to be weighed against laboratory costs, which increase with hare density.

## CONFLICT OF INTEREST

None declared.

## DATA ACCESSIBILITY

Microsatellite data are available for download at http://datadryad.org under DRYAD Repository entry https://doi.org/10.5061/dryad.s04h8. Simulation R code is provided in Appendix S3.
