# Supplementary material for: Genetic sampling for estimating density of common species
Source: Ecol Evol. 2017 Jul 3;7(16):6210–9. doi: 10.1002/ece3.3137 (PMC5574800; doi:10.1002/ece3.3137)
Supplement: Supplementary file 1 [file ECE3-7-6210-s001.docx]

**Supporting Information**

Additional Supporting Information may be found in the online version of this article.

**Appendix S1.** Captures and proportion recaptures for selected simulation scenarios.

**Appendix S2.** Additional results from simulation study.

**Appendix S3.** Simulation R code.

**Appendix S1.** Captures and proportion recaptures for selected simulation scenarios.

Relationship between estimated density, number of unique individuals captured, and % recaptures, from simulations based on our snowshoe hare study system. Each point represents one iteration (out of 500) for the specified simulation scenario. The red horizontal lines indicate 'true' (simulated) density. Estimated density is generally unbiased and close to true density when at least 20 unique individuals are captured (colored points excluding red) and at least 20% of those individuals are recaptured (marked by the gray dashed vertical line).

Above, results for the simulation scenario g0=0.25, sigma=80, and D=1.8. These patterns were typical of simulated scenarios with low g0.

Above, results for the simulation scenario g0=0.25, sigma=80, and D=1.8. These patterns were typical of simulated scenarios with moderate to high g0 and sigma.

**Appendix S2.** Additional results from simulation study.

Root mean square error for simulations based on our snowshoe hare study system. For each of 5 sampling approaches (figure legend), we simulated three hare densities (0.2, 1.0, and 1.8 hares per hectare, in figure rows), three detection probabilities at activity center (g0 = 0.05, 0.10, and 0.15, in figure columns), and three levels of sigma (20, 50, and 80 meters, on x-axis in figure cells). If less than 10% of the 500 iterations could not be estimated for any scenario, results are left blank.

Coefficient of variation for simulations based on our snowshoe hare study system. For each of 5 sampling approaches (figure legend), we simulated three hare densities (0.2, 1.0, and 1.8 hares per hectare, in figure rows), three detection probabilities at activity center (g0 = 0.05, 0.10, and 0.15, in figure columns), and three levels of sigma (20, 50, and 80 meters, on x-axis in figure cells). For each simulated scenario, we present the median coefficient of variation of estimable iterations, defined as capture histories that included at least one recapture. If less than 10% of the 500 iterations could not be estimated for any scenario, results are left blank.

**Appendix S3.** Simulation R code.

install.packages("secr")

library(secr)

### SCR parameters to vary in simulations ###

sim.D <- c(0.2, 1.0, 1.8) # density, in hares per hectare

sim.g0 <- c(0.05, 0.10, 0.15) # scr parameter g0

sim.sigma <- c(20, 50, 80) # scr parameter sigma, in meters

# --------------------------------------------------------------- #

# ----- SIMULATE LIVE-TRAPPING ----- #

# --------------------------------------------------------------- #

### Generate 500 iterations of simulated live-trapping capture history ###

temptrap.LT <- make.grid(nx=8, ny=10, spacing=50, detector="single") # make 8X10 trap grid, with 50-meter spacing between traps

sim.LT.CH <- list()

for(d in sim.D) { # for each density

for(g in sim.g0) { # for each g0

for(s in sim.sigma) { # for each sigma

for (rep in 1:500) {

sim.LT.CH[[paste0("D", d, "g", g, "s", s, "_", rep)]] <- sim.capthist(traps=temptrap.LT, popn=sim.popn(D=d, core=temptrap.LT, buffer=600), detectpar=list(g0=g, sigma=s), noccasions=4) # assumes four trap-nights

} # end rep loop

} # end s loop

} # end g loop

} # end d loop

### Estimate simulated live-trap secr parameters. Note that multi-catch likelihood is used for single-catch traps (explained in 'secr' package manual) ###

sim.LT.out <- list()

for(i in 1:length(sim.LT.CH)) {

LT.mask <- make.mask(traps(sim.LT.CH[[i]]), buffer = 600, type = 'trapbuffer')

possibleError <- sim.LT.CH[[i]][1] # error handling

if(!is.na(possibleError)){ # skip if no captures at all

sim.LT.out[[names(sim.LT.CH)[[i]]]] <-secr.fit(sim.LT.CH[[i]], model=list(D~1, g0~1, sigma~1), mask=LT.mask, start=list(D=1.2, g0=.15, sigma=65), method = "Nelder-Mead", trace = FALSE) # start values are means of simulated ranges

} # end if no captures

} # end i loop

# --------------------------------------------------------------- #

# ----- SIMULATE NGS ----- #

# --------------------------------------------------------------- #

### Generate 500 iterations of simulated NGS capture history. Code is presented for sampling scenario of 160 NGS plots and genotyping up to 2 pellets per NGS plot. This code can be modified to simulate a different number of plots (e.g., 80 NGS plots) and for genotyping fewer or more pellets per NGS plot. ###

temptrap.pell.all <- make.grid(nx=8, ny=20, spacex=50, spacey=25, detector="count") # make 8X20 NGS grid (for total of 160 NGS plots)

sim.pell.all.CH <- list()

for(d in sim.D) { # for each density

for(g in sim.g0) { # for each g0

for(s in sim.sigma) { # for each sigma

for (rep in 1:500) {

sim.pell.all.CH[[paste0("D", d, "g", g, "s", s, "_", rep)]] <- sim.capthist(traps=temptrap.pell.all, popn=sim.popn(D=d, core=temptrap.pell.all, buffer=600), detectpar=list(g0=g, sigma=s), noccasions=1) # single-occasion NGS

} # end rep loop

} # end s loop

} # end g loop

} # end d loop

### Estimate simulated NGS secr parameters, assuming up to 2 pellets genotyped per plot. ###

sim.pell2.CH <- sim.pell2.out <- list()

for (i in 1:length(sim.pell.all.CH)) {

if(nrow(sim.pell.all.CH[[i]])>0) { # skip if no captures at all

# from each plot, "sample" up to two pellets to genotype...

z<-data.frame(cbind(session=1, ID = as.numeric(animalID(sim.pell.all.CH[[i]])), occasion = 1, Trap = trap(sim.pell.all.CH[[i]]))) # convert capture format

z$session <- as.integer(z$session)

z$occasion <- as.integer(z$occasion)

z <- merge(z, temptrap.pell.all, by.x="Trap", by.y=0, all.x=TRUE)

pell2 <- lapply(split(z, z$Trap),

function(x) x[sample(1:nrow(x), min(nrow(x), 2)), ])

pell2 <- data.frame(Reduce(rbind, pell2))

pell2b <- pell2

pell2b$Trap <- NULL

sim.pell2.CH.count <- make.capthist(pell2b, temptrap.pell.all, fmt='XY', noccasions=1)

sim.pell2.CH[[i]] <- reduce(sim.pell2.CH.count, outputdetector='proximity') # createsim the "subset" capture history. Notice that detector is 'proximity' because if the same genotype appears more than once on an NGS plot, the animal can only be counted once for that plot.

pell2 <- unique(pell2)

pell2.mask <- make.mask(traps(sim.pell2.CH[[i]]), buffer = 600, type = 'trapbuffer')

possibleError <- sim.pell2.CH[[i]][1] # error handling

if(!is.na(possibleError)) {

sim.pell2.out[[names(sim.pell.all.CH)[[i]]]] <-secr.fit(sim.pell2.CH[[i]], model=list(D~1, g0~1, sigma~1), mask=pell2.mask, start=list(D=1.2, g0=.15, sigma=65), method = "Nelder-Mead", trace = FALSE) # the start values are the means of the simulated ranges

}

}

}
